# Clinical Outcomes of Partial Two-Stage Revision with Femoral Stem Retention in Elderly Patients with Hip Periprosthetic Joint Infection

**DOI:** 10.3390/jcm15062102

**Published:** 2026-03-10

**Authors:** Ji Hoon Bahk, Jun-Dong Chang, Young Wook Lim, Sinje Kim, Kee-Haeng Lee

**Affiliations:** 1 Department of Orthopedic Surgery, Bucheon St. Mary’s Hospital, College of Medicine, The Catholic University of Korea, Seoul 06591, Republic of Korea; tonybahk@gmail.com (J.H.B.);; 2 Department of Orthopedic Surgery, Hallym University Dongtan Sacred Heart Hospital, Hwaseong-si 18450, Republic of Korea; 3 Department of Orthopedic Surgery, Seoul St. Mary’s Hospital, College of Medicine, The Catholic University of Korea, Seoul 06591, Republic of Korea

**Keywords:** prosthesis-related infections, hip arthroplasty, reoperation, aged, treatment outcome

## Abstract

**Background/Objectives**: Periprosthetic joint infection (PJI) of the hip remains a challenging complication, particularly in elderly patients who may not tolerate repeated invasive procedures. While two-stage, one-stage, and 1.5-stage revisions are established strategies, an optimal approach for elderly patients with a well-fixed femoral stem remains unclear. This study evaluated the clinical outcomes of partial two-stage revision with femoral stem retention in elderly patients with hip PJI. **Methods**: A retrospective review was conducted of patients aged 65 years or older who underwent two-stage revision for hip PJI without femoral stem extraction at a single institution and had a minimum follow-up of one year after the final treatment. Patients were treated with aggressive debridement, removal of all components except the femoral stem, and placement of an antibiotic-loaded cement spacer and beads. Clinical outcomes, infection eradication, complications, and functional status were assessed. **Results**: Twenty-eight patients (28 hips) were included, with a mean age of 79.5 years and a mean follow-up of 46.2 months. The index arthroplasty was hemiarthroplasty in most cases (92.9%). Seventeen patients completed a second-stage revision, while 11 remained with cement spacer retention. Infection control was achieved in all patients (100%) at final follow-up, with initial infection control achieved in 96.4%. No recurrence of infection was observed in either group. Multidrug-resistant organisms were identified in 67.9% of cases. Functional outcomes were acceptable, and no stem-related complications or reinfections occurred. **Conclusions**: Partial two-stage revision with femoral stem retention provided effective infection control and acceptable functional outcomes in elderly patients with hip PJI. This approach may be considered a reasonable treatment option for elderly patients with a well-fixed femoral stem when reducing surgical invasiveness is an important consideration.

## 1. Introduction

Periprosthetic joint infection (PJI) is one of the most challenging complications following total hip arthroplasty (THA) or bipolar hemiarthroplasty. It is associated with substantial morbidity, prolonged treatment, and increased healthcare costs. The primary goal of PJI management is eradication of bacterial biofilm on prosthetic surfaces while restoring limb function and preserving quality of life. This dual objective makes surgical treatment of PJI particularly complex.

Traditionally, two-stage revision has been regarded as the gold standard for the management of chronic PJI [1,2,3,4,5,6,7,8,9]. This approach involves removal of the prosthesis and placement of an antibiotic spacer during the first stage, followed by definitive conversion to THA after infection control. Although high eradication rates have been reported, the requirement for two major procedures often delays rehabilitation and imposes substantial physiological stress, particularly in elderly patients. To address these limitations, one-stage revision has emerged as an alternative in selected patients [10,11,12,13,14,15,16,17,18]. This approach offers advantages including shorter treatment duration, faster functional recovery, and reduced morbidity. However, its application is restricted to carefully selected cases, typically involving known and susceptible pathogens, favorable soft tissue conditions, and healthy hosts with adequate physiological reserves. Consequently, one-stage revision is generally limited to acute infections and selected low-risk patients.

In elderly patients with multiple comorbidities, repeated surgical interventions may carry substantial risks, necessitating alternative strategies that balance infection control with surgical burden. In this context, the concept of a 1.5-stage revision has been introduced [19,20,21,22,23]. This strategy involves complete implant removal followed by reconstruction using cemented components, with the intention of avoiding a second planned procedure. However, removal of both acetabular and femoral components remains invasive and may still be demanding for older patients.

Given these challenges, partial two-stage revision has been proposed as another alternative approach [24,25,26,27,28,29,30,31,32]. This technique retains a well-fixed femoral stem, and in some cases the acetabular component, during staged revision. By preserving stable components, surgical invasiveness is reduced, potentially lowering perioperative risk and facilitating earlier functional recovery. This approach may be particularly suitable for patients aged 65 years or older with well-fixed femoral stems who are unlikely to tolerate extensive revision surgery. The purpose of this study was to evaluate the clinical outcomes of elderly patients treated with partial two-stage revision for hip PJI with femoral stem retention, focusing on infection eradication, functional outcomes, and patient survival.

## 2. Materials and Methods

### 2.1. Patient Cohort and Characteristics

This study was approved by the institutional review board of the study institution (HC23RISI0154) before initiation. Medical records and hip radiographs were retrospectively reviewed for all two-stage revision hip surgeries performed without extraction of a well-fixed femoral stem for bacterial PJI over a 16-year period, from January 2008 to December 2024, at a single institution. Patients aged 65 years or older at the time of PJI diagnosis who had complete clinical records with a minimum follow-up of one year after the final revision surgery were included. The mean follow-up duration was 46.2 months (range, 12 to 159 months). All revision procedures following the diagnosis of PJI were performed by a single high-volume senior hip arthroplasty surgeon. Cases of native joint infection, periprosthetic infection involving orthopedic hardware other than arthroplasty components, revisions with complete implant removal including femoral stem extraction, and one-stage or 1.5-stage revision procedures involving cemented implants were excluded from the study.

A total of 28 patients (28 hips) were included for analysis, including 26 women (92.9%) and two men (7.1%), with a mean age at the time of first revision surgery of 79.5 ± 5.7 years (range, 66 to 90) (Table 1). Most index arthroplasties were hemiarthroplasties (92.9%). The most commonly identified previously implanted femoral components at the time of PJI diagnosis were Bencox II^®^ (Corentec, Seoul, Republic of Korea) (42.9%), followed by Summit^®^ (DePuy Orthopaedics Inc., Warsaw, IN, USA) (25.0%), with fully or near-fully coated stems accounting for 85.7% of cases. Detailed clinical and infection-related characteristics of each patient are summarized in Table 2.

### 2.2. Diagnosis of PJI and Selection of Femoral Stem Retention

The 2018 Musculoskeletal Infection Society (MSIS) criteria were retrospectively applied to all cases with a confirmed diagnosis of PJI [33]. To identify the causative pathogen, blood cultures were obtained for all patients at the time of initial evaluation, and intra-articular aspiration was attempted. To assess proximal femoral involvement, in addition to plain radiographs, preoperative bone scintigraphy and magnetic resonance imaging were routinely obtained. In addition, intraoperative frozen biopsy to assess the neutrophil count per high-power field was requested, and specimens for culture and permanent pathology were obtained intraoperatively (Table 3).

The decision to retain or remove the femoral stem was based on preoperative radiographic assessment and intraoperative findings. In cases of cementless stems demonstrating radiographic evidence of bone ingrowth limited to the proximal one third or less, with radiolucent lines around the distal portion of the stem [34,35], an attempt was made to remove the stem by gentle extraction using a stem remover. If stem removal was unsuccessful using this technique, the femoral component was retained. In contrast, when preoperative radiographs demonstrated bone ingrowth along the entire length of the stem, stem removal was not attempted. On bone scintigraphy, uptake confined to the proximal femoral entry region was considered acceptable for stem retention, whereas any uptake extending along the femoral canal adjacent to the femoral component precluded stem retention.

Intraoperatively, femoral stems were considered osseointegrated if they could not be removed without the aid of osteotomy. Stem retention was further considered only in the absence of gross proximal femoral defects communicating with the distal femoral canal on direct inspection or curettage. Based on these criteria, all acetabular components were removed during the first-stage radical debridement, whereas the femoral component was retained in selected cases. In one patient, the femoral stem was removed due to suspected osteolysis and was therefore excluded from the stem-retention cohort. Partial exchange revision was performed only when the femoral stem was confirmed to be well fixed.

### 2.3. Surgical Technique of First-Stage Revision with Stem Retention

The first stage consisted of aggressive open surgical debridement through a posterior approach, during which multiple deep tissue specimens were obtained for microbiological analysis. All prosthetic components except the femoral stem were removed, followed by extensive irrigation with normal saline. When an acetabular cup was present, it was routinely removed, and the acetabular bed was reamed to disrupt residual biofilm. In cases of hemiarthroplasty, the remaining acetabular cartilage was completely reamed. A gentamicin-premixed polymethyl methacrylate bone cement product (CMW^®^, DePuy Orthopaedics Inc., Warsaw, IN, USA) was mixed with 1 g of vancomycin per 40 g (one pack) of cement powder, with two packs generally used, to create the cement spacer and beads. The cement spacer was shaped into a near-spherical geometry with greater-than-hemispherical coverage, with a new ceramic head embedded centrally to allow engagement with the femoral component taper [24]. Cement beads were hand-molded into spherical shapes measuring 8 to 9 mm in diameter and assembled into strings, each consisting of 6 to 8 beads. In total, 16 to 30 beads were inserted into the intracapsular space and, when a draining sinus was present, the sinus tract was completely excised, and additional beads were placed around the greater trochanter near the site of the previous draining sinus.

### 2.4. Postoperative Monitoring and Second-Stage Revision

Postoperatively, patients were mobilized with full weight-bearing as tolerated under physiotherapy supervision. Antibiotic therapy was maintained according to microbiological findings and clinical response. Clinicalal and radiological monitoring was performed at regular outpatient follow-up visits, with assessment of wound healing and laboratory tests including inflammatory markers. Infection control was evaluated after a six-week course of antibiotic therapy.

Repeated DAIR procedures were considered if eradication failed and patients demonstrated gross inflammatory findings or recurrent elevation of inflammatory markers accompanied by fever. The second stage of revision typically occurred within three months and involved removal of the cement spacer, additional tissue sampling for frozen section analysis to assess the neutrophil count under high-power field microscopy, followed by definitive conversion to cementless THA if infection control was confirmed. In a limited number of patients, the second-stage procedure was not performed, and ambulation was permitted with the cement spacer in situ. Postoperatively, patients were mobilized to full weight-bearing as tolerated and enrolled in a physiotherapy-based rehabilitation program as early as possible, including patients with a cement spacer in situ.

### 2.5. Perioperative Antibiotic Management

Empiric vancomycin was selected as the initial antibiotic to provide coverage against common causative organisms of periprosthetic joint infection, including MRSA, MRCoNS, and Enterococcus species [36]. Subsequent antibiotic selection and modification were guided by antibiogram results in consultation with infectious diseases specialists. All decisions regarding oral step-down therapy were made through multidisciplinary discussion. An identical antibiotic protocol was applied in patients managed with cement spacer retention.

Intravenous antibiotic therapy was generally administered for 6–8 weeks following the initial procedure, after which the second-stage operation was performed. In cases in which the second-stage procedure was delayed due to other medical conditions, oral antibiotics were continued as suppressive therapy based on prior susceptibility results. For infections caused by MRSA or MRCoNS, vancomycin was used as the primary agent, with teicoplanin selected as an alternative first-line agent in cases showing greater susceptibility on antibiogram or when vancomycin use was limited by renal impairment. Patients who developed vancomycin-related adverse events, including nephrotoxicity or neutropenia, were switched to teicoplanin. In cases of ESBL-producing Escherichia coli, carbapenem-based regimens were primarily administered.

At the time of the second-stage operation, intravenous antibiotics used during the initial stage were generally continued. Patients were typically discharged after approximately two weeks of postoperative antibiotic therapy when there was no evidence of persistent infection. Upon discharge, additional oral antibiotics were prescribed for 2–6 weeks based on the patient’s general condition and microbiological findings. Prolonged suppressive antibiotic therapy beyond this period was not routinely used in the absence of other medical indications. Only four patients required continuation beyond six weeks due to concomitant conditions, including bacterial peritonitis, cholangiocarcinoma, pneumonia, or recurrent urinary tract infection.

During oral step-down therapy, trimethoprim–sulfamethoxazole, fluoroquinolones, or cephalosporins were selected according to susceptibility results. In selected cases, rifampicin was added to the oral regimen after an adequate course of intravenous therapy. In cases showing no neutrophils on intraoperative frozen biopsy during the second-stage procedure, with no serological or radiological evidence of ongoing infection, no additional oral antibiotics were administered.

### 2.6. Clinical and Radiological Evaluation of Outcomes

The primary outcome of this analysis was infection eradication, assessed by the presence of recurrence of infection or uncontrolled infection after the first revision surgery. In patients managed with cement spacer retention, successful treatment was defined as infection control, acknowledging the potential presence of dormant bacteria. Other surgical complications identified during the follow-up period were also investigated. After the first revision surgery, infection was considered controlled when the following criteria were met: absence of clinical, serological findings including normalized serum C-reactive protein (CRP) levels (0–5 mg/L), and radiographic signs of infection, and no mortality secondary to infection or its treatment during the follow-up period after the final treatment, which could be definitive THA or cement spacer retention. Serum erythrocyte sedimentation rate (ESR) levels were monitored as a supplementary inflammatory marker during follow-up but were not required to normalize for the determination of infection control, as ESR may increase with age [37], while serum C-reactive protein (CRP) was used as the primary serological indicator in this predominantly elderly cohort with chronic comorbidities [38].

Clinical and radiographic follow-up evaluations were performed at 2 and 6 weeks after surgery, at 3, 6, 9, and 12 months postoperatively, and every 6 months thereafter. Patients who were lost to follow-up were contacted by telephone and encouraged to return for outpatient evaluation. The Harris Hip Score (HHS; range, 0 to 100) was assessed in all patients. However, interpretation of HHS was limited in a small number of patients with severe baseline ambulatory impairment, including those with a Koval grade of 7 [39], in whom reliable functional assessment was not feasible. Pain intensity was evaluated using a numeric rating scale (NRS) at follow-up visits. The presence of prosthesis-related symptoms including squeaking and anterior thigh pain was routinely assessed. Femoral component loosening, osteolysis, wound dehiscence, hematoma formation, deep vein thrombosis, pulmonary thromboembolism, dislocation, and periprosthetic fracture were evaluated at each follow-up visit.

## 3. Results

Among the included patients, 17 of 28 completed the second-stage procedure, while 11 did not proceed beyond the first-stage revision with cement spacer insertion. Reasons for not proceeding to the second stage despite successful infection control were most commonly patient or legal guardian refusal due to reluctance, poor general condition, or financial concerns (72.7%), followed by recovery of preoperative ambulatory status (18.1%) (Table 4).

The overall infection control rate was 100% (28 of 28), with no recurrence of infection at the final follow-up. According to the predefined outcome definitions, infection eradication was applicable only to patients who completed second-stage revision and was achieved in all such cases (100%, 17 of 17). When the need for a repeat first-stage procedure was considered, initial infection control was achieved in 96.4% of patients (27 of 28). Among patients who proceeded to definitive surgery after infection control, the mean interval between the first-stage revision and definitive THA was 13.6 weeks, and no recurrence was observed during a mean infection-free survival of 38.4 months (3.2 years). Eleven patients maintained the cement spacer as the final treatment, with a mean infection-free survival of 51.2 months (4.3 years) after the last surgery until the final follow-up (Table 5). The interval from index surgery to first revision was longer in the two-stage completed group compared with the cement spacer retention group (50.1 vs. 9.9 weeks). This difference was primarily attributable to cases of very late-onset PJI, with infections occurring at 54 and 89 months after the index arthroplasty in the two-stage completed group.

The follow-up rate was 75.0% (21 of 28) when deceased patients were excluded from loss to follow-up and 35.7% (10 of 28) when deceased patients were included as lost to follow-up. During the follow-up period, 6 patients were lost to follow-up, 12 patients died, and 10 patients remained under active follow-up. All deaths were confirmed to be unrelated to PJI. The mean survival time among deceased patients was 31.4 months, corresponding to their follow-up duration.

One patient required an additional round of debridement and cement spacer exchange 12 weeks after the first revision because serologic inflammatory markers failed to normalize. Infection was subsequently controlled, but the development of pulmonary tuberculosis led to deterioration of the general condition, which did not allow definitive conversion to THA.

Regarding complications, one patient who underwent definitive second-stage revision sustained a Vancouver type B1 periprosthetic fracture near the distal tip of the stem three months after surgery following a fall, which was treated with open reduction and internal fixation using a plate and screws. Six months later, the same patient sustained a Vancouver type C periprosthetic fracture near the distal end of the previously inserted plate, which was treated with revision internal fixation using a longer plate. Despite these events, no recurrence of infection was observed.

In another patient, infection was controlled with cement spacer retention, and the patient was able to ambulate with a walker. Several weeks later, ambulation was discontinued because of knee pain due to advanced osteoarthritis. In addition, acetabular erosion with medial migration of the cement spacer by 8 mm was observed. Definitive THA and total knee arthroplasty were recommended, but the patient and legal guardians declined further surgery.

No squeaking, anterior thigh pain, femoral component loosening, osteolysis, wound dehiscence, hematoma formation, deep vein thrombosis, pulmonary thromboembolism, or dislocation was observed throughout the follow-up period. The mean Harris Hip Score at final follow-up was 88.0 ± 12.1 in patients who completed second-stage surgery (n = 17) and 64.9 ± 19.4 in those managed with cement spacer retention (n = 9), and the mean NRS pain score at final follow-up was 1.6 ± 1.8 and 2.9 ± 2.0, respectively. Two patients were excluded from the HHS analysis due to inability to perform reliable functional assessment.

## 4. Discussion

Traditional two-stage revision, one-stage revision in selected healthy patients, and the more simplified 1.5-stage approach have expanded the available surgical options for the management of PJI. In elderly patients, however, reducing the invasiveness of each surgical step becomes an important consideration, particularly when multiple procedures are anticipated. In this context, even avoiding a single aggressive step may meaningfully reduce surgical burden. Removal of infected components is fundamental to infection control. Acetabular cup removal is generally less demanding, whereas extraction of a femoral stem is often considerably more challenging. Although techniques such as extended trochanteric osteotomy or trephine reaming can be used to facilitate stem removal, these procedures remain aggressive and are associated with considerable blood loss and substantially prolonged operative time. Therefore, in elderly patients with a well-fixed femoral stem, a partial two-stage strategy with femoral stem retention can be a reasonable option.

When interpreting the infection eradication rate observed in the present study, it is important to consider these results in the context of previously published literature. As summarized in Table 6, studies reporting fewer than 10 cases of partial two-stage revision have occasionally demonstrated eradication rates of 100%. In contrast, reports including more than 10 patients have consistently shown eradication rates in the range of approximately 80% to 90%. Importantly, none of these previous studies restricted patient inclusion based on age. In this regard, the 100% eradication rate observed in the current series of 28 hips, which specifically focused on an elderly population, appears favorable when compared with prior reports of similar or larger cohort size. Although direct comparisons are limited by heterogeneity in patient characteristics, infection chronicity, and surgical technique, these findings suggest that partial two-stage revision with femoral stem retention may achieve acceptable infection control in elderly patients. Rather than implying superiority, our results support the view that this strategy can be considered when minimizing surgical invasiveness is a priority.

In the present cohort, late chronic infection accounted for the majority of cases, and a high proportion of multidrug-resistant (MDR) organisms was identified. Even after excluding polymicrobial infections, multidrug-resistant pathogens were isolated in 67.9% of patients, including methicillin-resistant coagulase-negative *staphylococci* (MRCoNS), methicillin-resistant *Staphylococcus aureus* (MRSA), vancomycin-susceptible *Enterococcus* (VSE), and a case of extended-spectrum beta-lactamase–producing (ESBL) *Escherichia coli*. Despite this unfavorable microbiologic profile, positive bloodstream infection was documented in only 39.3% of cases. When stratified by infection chronicity, multidrug-resistant organisms were identified in 75.0% of acute, 66.7% of subacute, and 62.5% of late infections, with no substantial difference according to the timing of infection. Infection control of the hip joint was nonetheless achieved in all patients, suggesting that early local infection management and surgical strategy may play a meaningful role even in the presence of resistant organisms.

In acute presentations, our treatment strategy favored early surgical intervention rather than prolonged conservative management or DAIR. Although DAIR is often considered a less invasive option, it still represents a meaningful surgical insult and may still be demanding for patients, particularly in elderly patients. For this reason, when the clinical course or host condition did not appear clearly favorable, we proceeded directly to a first-stage revision rather than attempting DAIR. When PJI is identified early and managed surgically without delay, many femoral stems remain well fixed. In the present series, no case underwent partial revision with acetabular component retention, and only one case during the same study period required femoral stem extraction. Although this may reflect a degree of selection bias inherent to our patient population, the high proportion of fully or near-fully coated stems may have contributed to stem stability. Even in proximally coated stems, sufficient metaphyseal osseointegration may allow safe stem retention. Furthermore, although the concept of an effective joint space is relevant, meticulous debridement followed by circumferential sealing with antibiotic-loaded cement may adequately address this concern in selected cases. Confidence in this approach may be further supported when no increased uptake is observed in the diaphyseal portion of the femur on preoperative bone scan.

From a functional perspective, patients who remained with a cement spacer after the first-stage procedure demonstrated outcomes that were often better than anticipated. Although ambulation with a spacer was not routinely encouraged, several patients maintained acceptable mobility with only minimal decline in Koval grade and limited discomfort. Some patients were able to ambulate outdoors with a cane. In patients with limited baseline mobility, preservation of joint range of motion and tolerance of basic rehabilitation appeared favorable compared with conventional spacers. Functional score comparisons between groups should be interpreted with caution, as patients managed with cement spacer retention often had limited baseline ambulatory capacity, including individuals with a preoperative Koval grade of 7, for whom conventional hip-specific functional scores may have limited interpretability.

In addition, retention of the femoral taper allows use of a ceramic head, which provides structural durability without unnecessary cement bulk. Given that the core volume of a cement spacer contributes little to antibiotic elution, this design may represent a practical balance between mechanical stability and local antimicrobial delivery. Nevertheless, cement spacer retention, whether planned or unplanned, should be regarded as a compromise strategy rather than definitive treatment. Prolonged use of a cement spacer in patients with higher activity levels may result in acetabular erosion. To minimize this risk, careful shaping of the cement spacer into a smooth and spherical contour at the time of fabrication, along with regular radiographic follow-up, is advised to monitor acetabular wear. Progressive acetabular erosion or worsening symptoms during follow-up may ultimately necessitate conversion to a second-stage procedure.

**Figure 1:**
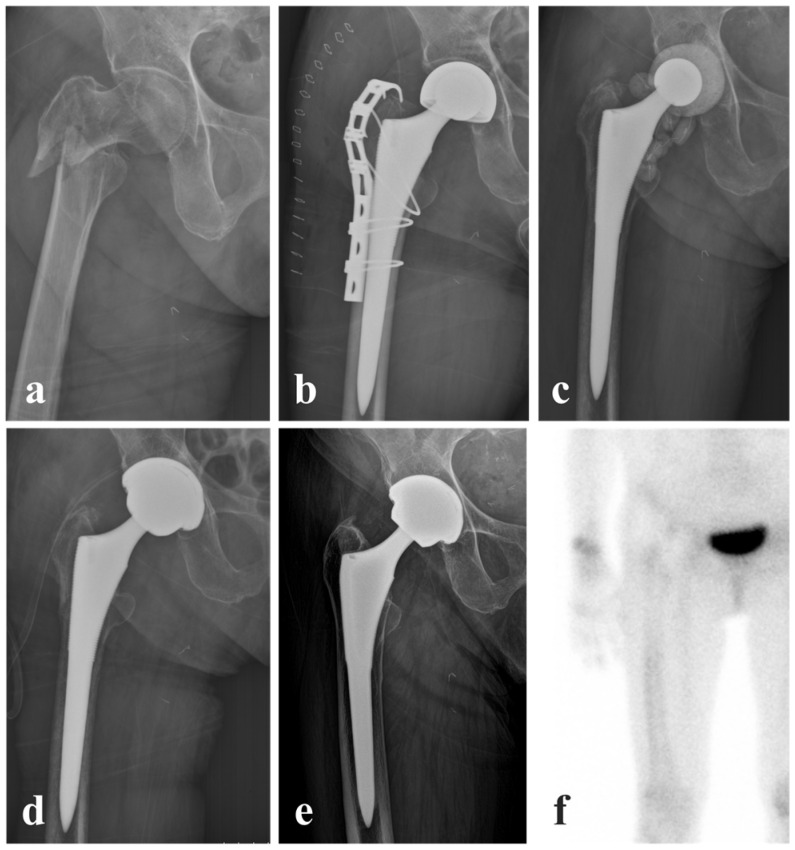
A case of a 74-year-old female treated with complete two-stage revision with femoral stem retention. (**a**) Preoperative radiograph showing an intertrochanteric fracture. (**b**) Postoperative radiograph after index arthroplasty with an additional trochanteric reattachment device. (**c**) Postoperative radiograph after the first-stage revision with femoral stem retention. (**d**) Immediate postoperative radiograph after the second-stage revision with definitive THA. (**e**) Follow-up radiograph obtained 13.3 years postoperatively. (**f**) Bone scan image obtained 10 years after surgery, showing negative uptake around the right hip joint.

Two representative cases are presented to illustrate the clinical outcomes of femoral stem retention during complete or partial two-stage revision, in which infection control was achieved in both cases, with definitive conversion to THA limited to the complete two-stage revision. A 74-year-old female underwent hemiarthroplasty with additional grip plate fixation after a fall resulting in a comminuted intertrochanteric fracture (Figure 1). Her medical history included diabetes mellitus, hypertension, and right-sided hemiplegia with motor grade IV following cerebral infarction, and she was receiving aspirin therapy. She remained hospitalized for four weeks for recovery of her general condition and was discharged in a stable condition. At three months postoperatively, the patient presented with gross clinical signs of inflammation and elevated inflammatory markers, including an erythrocyte sedimentation rate of 44 mm/h and a C-reactive protein level of 20.68 mg/L. Blood culture and joint aspiration culture were negative. However, an elevated D-dimer level, apparent intraoperative purulence, increased synovial white blood cell count with a high polymorphonuclear neutrophil percentage, and positive histologic findings led to the diagnosis of PJI. The patient received intravenous vancomycin and underwent two-stage revision with femoral stem retention, with a seven-week interval between the first- and second-stage procedures. At the most recent follow-up, 13.3 years after surgery, the patient remained satisfied with the outcome and was able to ambulate independently without evidence of recurrent infection or pain.

**Figure 2:**
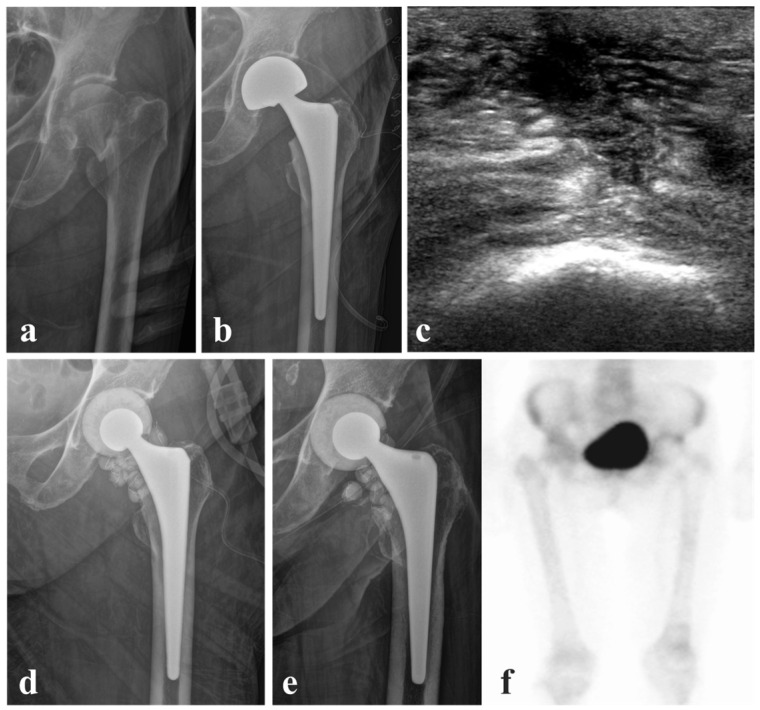
A case of a 78-year-old patient treated with partial two-stage revision that was discontinued after the first revision. (**a**) Preoperative anteroposterior hip radiograph showing a femoral neck fracture after a fall. (**b**) Postoperative radiograph after bipolar hemiarthroplasty. (**c**) Sonographic image obtained before aspiration, suggestive of PJI. (**d**,**e**) Immediate postoperative and 2-year follow-up radiographs after the first-stage revision with femoral stem retention. (**f**) Bone scan image obtained 2 years after surgery, showing negative uptake around the left hip joint.

In contrast, a 78-year-old female underwent bipolar hemiarthroplasty for a femoral neck fracture after tripping while ambulating with a walker in a long-term care facility (Figure 2). Her medical history included diabetes mellitus, hypertension, stable angina, asthma, vascular parkinsonism, liver cirrhosis, and heart failure. After 14 days of inpatient care, she was discharged in a stable condition. The patient returned to the outpatient clinic one week after discharge, presenting with gross inflammatory signs including edema, erythema, and a sensation of heat around the incision site, accompanied by elevated inflammatory markers. Blood cultures were negative, whereas joint aspiration culture revealed oxacillin-resistant, vancomycin-susceptible *Staphylococcus capitis* (MRCoNS), and intravenous vancomycin therapy was initiated. The hemiarthroplasty shell and ceramic head were removed with retention of the femoral stem and replaced with a cement spacer and antibiotic-loaded beads. At four weeks postoperatively, both erythrocyte sedimentation rate and C-reactive protein levels had returned to the normal range. Because the patient declined further surgery, the second-stage procedure was not performed. At the two-year follow-up, the patient remained free of infection and was able to ambulate with a walker without discomfort, although radiographic evaluation demonstrated mild migration toward the acetabular side.

This study has several limitations. First, the retrospective design and small sample size, related in part to the relative rarity of PJI, limit the strength of the analysis. Although data were collected over an extended period, the sample size remains limited, which may affect statistical power. Second, a substantial proportion of the study population died from causes unrelated to the prosthesis, which complicates assessment of the direct impact of PJI and its treatment on overall clinical outcomes. Mortality related to comorbid conditions may have influenced the interpretation of PJI-related outcomes. Third, the study population consisted predominantly of elderly patients (mean age, 79.5 years) with multiple comorbidities, which was associated with a high likelihood of loss to follow-up due to death. Accordingly, the reported infection eradication rate of 100% and the observed period of infection control should be interpreted with caution, given the high attrition rate and competing risks of death in this cohort. As a result, follow-up duration varied substantially among patients. Lastly, the predominance of hemiarthroplasty in the study cohort limits the generalizability of the results. Outcomes observed in patients with hemiarthroplasty may differ from those in patients who underwent THA, and further studies with a greater proportion of THA cases may be needed. However, given the advanced age of the study population, a higher proportion of hemiarthroplasty was unavoidable.

## 5. Conclusions

Partial two-stage revision with femoral stem retention achieved reliable infection control in elderly patients with hip PJI, even in the presence of multidrug-resistant organisms. By avoiding femoral stem extraction, this approach reduced surgical invasiveness while maintaining acceptable functional outcomes. In selected elderly patients with a well-fixed femoral stem, partial two-stage revision may serve as a practical treatment option when minimizing surgical burden is a priority.

## Figures and Tables

**Table 1:** Patient Demographics and Clinical Characteristics.

Parameter	Total (%)	Two-Stage Completed	Cement Spacer Only
Age at diagnosis (years)	79.50 ± 5.67	79.5 (66–89)	79.5 (69–90)
Sex			
Male	2 (7.1)	1	1
Female	26 (92.9)	16	10
Type of index arthroplasty			
Hemiarthroplasty	26 (92.9)	17	9
Total hip arthroplasty	2 (7.1)	0	2
Site of index arthroplasty			
This institute	13	12	10
Referred from other hospital	15	5	1
Classification of PJI by timing			
Acute (<4 weeks)	8 (28.5)	4	4
Subacute (4 weeks to 3 months)	12 (42.9)	7	5
Late (>3 months)	8 (28.5)	6	2
Positive blood culture at the time of diagnosis			
Yes	11 (39.3)	6	5
No	17 (60.7)	11	6
Prior treatment before first revision surgery			
DAIR procedure	3 (10.7)	3	0

PJI indicates periprosthetic joint infection; DAIR for debridement, antibiotics, and implant retention.

**Table 2:** Clinical and infection-related characteristics of included patients.

Patient Number	Final Tx.	Index Surgery	Stem Manufacturer	Infection Type	Prior Tx.	Pathogen	Blood Culture
1	THA	Cementless HA	Bencox II^®^	Late	None	MSSE	(−)
2	THA	Cementless HA	Replica^®^	Subacute	DAIR	MRCoNS	(+)
3	THA	Cementless HA	Bencox II^®^	Acute	None	Culture negative	(−)
4	THA	Cementless HA	Summit^®^	Acute	None	MRCoNS	(−)
5	THA	Cementless HA	Bencox II^®^	Acute	None	MRCoNS	(−)
6	THA	Cementless HA	Summit^®^	Late	None	VSE	(−)
7	THA	Cementless HA	AML^®^	Subacute	None	Culture negative	(−)
8	THA	Cementless HA	Summit^®^	Subacute	None	Culture negative	(−)
9	THA	Cementless HA	Bencox II^®^	Acute	DAIR	MRSA	(+)
10	THA	Cementless HA	Opti-Fix^®^	Late	None	MRSA	(−)
11	THA	Cementless HA	Taperloc^®^	Late	None	MRCoNS, VSE	(−)
12	THA	Cemented HA	Total hip system^®^	Subacute	None	MRSA	(+)
13	THA	Cementless HA	Summit^®^	Subacute	None	MRSA	(−)
14	THA	Cementless HA	Summit^®^	Subacute	None	VSE	(+)
15	THA	Cementless HA	Summit^®^	Subacute	None	MRCoNS	(+)
16	THA	Cementless HA	Bencox II^®^	Late	None	Culture negative	(+)
17	THA	Cementless HA	Wagner SL^®^	Late	DAIR	MRCoNS	(−)
18	Spacer	Cementless HA	Bencox II^®^	Subacute	None	MRCoNS	(+)
19	Spacer	Cementless HA	Bencox II^®^	Acute	None	MRCoNS	(−)
20	Spacer	Cementless HA	Bencox II^®^	Subacute	None	MRCoNS	(−)
21	Spacer	Cementless HA	Bencox II^®^	Late	None	Corynebacterium spp.	(+)
22	Spacer	Cementless HA	Summit^®^	Subacute	None	ESBL(+) E.coli	(+)
23	Spacer	Cementless HA	Corail^®^	Subacute	None	Streptococcus spp.	(+)
24	Spacer	Cementless HA	Bencox II^®^	Subacute	Repeated stage-1	VSE	(−)
25	Spacer	Cementless HA	Bencox II^®^	Acute	None	MRCoNS, VSE	(−)
26	Spacer	THA	SOMA^®^	Late	None	MSSA	(+)
27	Spacer	THA cup revision	Opti-Fix^®^	Acute	None	MRSA	(−)
28	Spacer	Cementless HA	Bencox II^®^	Acute	None	MSSA	(−)

Tx. indicates treatment. Manufacturer information: Replica^®^, AML^®^, and Corail^®^ (DePuy Orthopaedics Inc., Warsaw, IN, USA); Opti-Fix^®^ (Smith & Nephew, Memphis, TN, USA); Taperloc^®^ and Wagner SL^®^ (Zimmer Biomet, Warsaw, IN, USA); Foundation Total Hip System^®^ (DJO Surgical, Austin, TX, USA); SOMA^®^ (Stryker, Mahwah, NJ, USA).

**Table 3:** Identified pathogens in PJI. Two cases had concomitant infection with methicillin-susceptible Staphylococcus epidermidis (MSSE) and vancomycin-susceptible Enterococcus (VSE).

Identified Pathogen	Count (%)
Methicillin-resistant coagulase-negative *Staphylococci* (MRCoNS)	10 (35.7)
Methicillin-resistant *Staphylococcus aureus* (MRSA)	5 (17.9)
Vancomycin-susceptible *Enterococci* (VSE)	5 (17.9)
Methicillin-susceptible *Staphylococcus aureus* (MSSA)	2 (7.1)
Methicillin-susceptible *Staphylococcus epidermidis* (MSSE)	1 (3.6)
Extended-spectrum β-lactamase (ESBL)-positive *Escherichia coli*	1 (3.6)
*Streptococcus* spp.	1 (3.6)
*Corynebacterium* spp.	1 (3.6)
Culture-negative	4 (14.3)

spp. indicates species.

**Table 4:** Functional Outcomes and Reasons for Not Proceeding to Second-Stage Surgery.

Patient Number	Preoperative Koval Grade	Postoperative Koval Grade	Reasons for Not Proceeding to Second-Stage Surgery
18	2	3	Patient refused further surgery; tolerated well
19	2	3	Achieved preoperative ambulatory status with satisfaction
20	3	3	Patient refused further surgery; high anesthetic risk
21	7	7	Achieved preoperative ambulatory status
22	5	6	Patient refused further surgery; high anesthetic risk
23	1	2	Patient refused further surgery
24	5	7	Deterioration of general condition due to pulmonary tuberculosis
25	1	2	Legal guardians refused further surgery
26	1	3	Legal guardians refused further surgery
27	6	6	Patient refused further surgery; relocation
28	2	3	Patient refused further surgery

**Table 5:** Infection-free survival and surgical interval.

Parameter	Total	Two-Stage Completed	Cement Spacer Only
Infection-free survival (months, *n* = 28)	46.2	38.4 (12–159)	51.2 (12–147)
Infection-free survival until death (months, *n* = 12)	52.75	69.0 (14–147)	44.6 (12–103)
Interval period			
Index surgery to first revision surgery (weeks)	34.3	50.1 (2–356)	9.9 (2–36)
First-stage revision to definitive THA conversion (weeks)	13.5	13.6 (4–49)	Not available *

Values are presented as mean or median (range). * Definitive THA conversion was not confirmed in this group.

**Table 6:** Previously published studies reporting partial two-stage revision.

Study	Published Year	Number of Hips	Infection Timing	Mean Follow-Up (Months)	Infection Eradicated or Controlled (%)
Lee et al. [24]	2013	19 (17 *)	Subacute-chronic	48	88.2
Ekpo et al. [25]	2014	19	Chronic	48	89
Fukui et al. [26]	2016	5	N/A	4.2	100
Chen et al. [27]	2017	16	Acute-chronic	70	81
Crawford et al. [28]	2019	41	N/A	5.5	81
Castagnini et al. [29]	2020	28	Chronic	60	83.4
Shi et al. [30]	2020	8	Chronic	67.4	100
Yishake [31]	2021	28	Subacute-chronic	48	85.7
Moreno-Romero et al. [32]	2023	8	Subacute-chronic	38.2	100
Current study	2026	28 (17 *)	Acute-chronic	46.2	100

* Values in parentheses indicate patients who completed partial two-stage revision and were eligible for the definition of infection eradication. Patients managed with cement spacer retention were considered to have achieved infection control.

## Data Availability

The datasets used and analyses are available from the corresponding author upon reasonable request.
